# Anxiety sensitivity, its stability and longitudinal association with severity of anxiety symptoms

**DOI:** 10.1038/s41598-019-39931-7

**Published:** 2019-03-13

**Authors:** Johanna H. M. Hovenkamp-Hermelink, Date C. van der Veen, Richard C. Oude Voshaar, Neeltje M. Batelaan, Brenda W.J.H. Penninx, Bertus F. Jeronimus, Robert A. Schoevers, Harriëtte Riese

**Affiliations:** 1 University of Groningen, University Medical Center Groningen (UMCG), Department of Psychiatry, Interdisciplinary Center Psychopathology and Emotional regulation (ICPE), Groningen, The Netherlands; 2 VU University Medical Center Amsterdam, Department of Psychiatry and Amsterdam Public Health Research Institute, Amsterdam, The Netherlands; 3 GGZ inGeest Amsterdam, Amsterdam, The Netherlands; 4 University of Groningen, Department of Developmental Psychology, Faculty of Behavioral and Social Sciences, University of Groningen, Groningen, The Netherlands

## Abstract

Anxiety sensitivity is associated with the onset of panic attacks, anxiety, and other common mental disorders. Anxiety sensitivity is usually seen as a relative stable trait. However, previous studies were inconclusive regarding the longitudinal stability of anxiety sensitivity and differed in study designs and outcomes. The current study examines the stability of anxiety sensitivity over time and its longitudinal associations with severity of anxiety symptoms. Participants from the Netherlands Study of Depression and Anxiety with and without an anxiety, depressive, or comorbid anxiety-depressive disorder diagnosis were included (*N* = 2052). Stability in anxiety sensitivity over two year follow-up and the longitudinal association between the change in anxiety sensitivity and change in severity of anxiety symptoms were tested. Results indicated that two-year stability of anxiety sensitivity was high (*r* = 0.72), yet this test-retest estimate leaves room for changes in anxiety sensitivity in some individuals as well. Change in anxiety sensitivity was positively associated with change in severity of anxiety symptoms (B = 0.64 in univariable analysis and B = 0.52 in multivariable analysis). The longitudinal association of anxiety sensitivity with severity of anxiety symptoms indicates that targeting anxiety sensitivity may be of additional benefit in clinical practice.

## Introduction

Anxiety sensitivity (AS) is a psychological risk factor that has received a lot of attention in clinical and epidemiological studies of anxiety disorders. AS can be described as the ‘fear of fear’ or the fear of anxiety-related symptoms such as heart palpitations, sweating, or shaking, due to the belief that these symptoms have negative consequences^1^. There is a lot of literature indicating that elevated AS levels predict the onset of anxiety symptoms and panic attacks^2,3^ as well as Axis I diagnoses, particularly anxiety and depressive disorders^4,5^. Furthermore, AS was found to be predictive of persistence of anxiety disorders in a four-year follow-up study in an earlier study in our sample^6^. Despite these findings, it remains unclear whether AS is also associated with the severity of anxiety symptoms over time, which is important, because higher severity of anxiety symptoms is associated with a poorer prognosis of anxiety and depressive disorder trajectories^7,8^.

Another question concerns the temporal stability of AS, which relates to the discussion whether AS can be distinguished from trait anxiety. Some scholars postulated that AS and trait anxiety are closely related and cannot be clearly distinguished from each other^9,10^. Others argue that AS and trait anxiety, although related, are different entities^11–14^ and called AS a ‘trait-like’ construct. If AS is akin to trait-anxiety AS should be equally stable over time. However, previous studies addressing the issue of AS stability over time were inconsistent. Reasonable AS stability was observed over a 44-week period (*r* = 0.72) in a study of 86 outpatients with one or more anxiety disorders^15^. Another study of 1277 high school students with four yearly assessments identified three subgroups with different AS stability trajectories^16^: A group with stable low (n = 1277), a group with stable high (n = 140) AS levels, and a groups with AS levels that increased over time (n = 320). Comparable results with three classes, that is normative-stable, high-stable, and high-unstable trajectories of AS, were reported by Allan *et al*.^17^. In addition, in an adolescents population a normative developmental change in AS was found over an on average two-year time period with a correlation of 0.47^18^. These longitudinal studies suggests that in some individuals AS can change substantially, a perspective that is in line with the results of a study in 1296 young adults attending intensive military training. In this healthy sample, AS was found to increase within five weeks in response to stressful physical and mental events that triggered anxiety symptoms and panic attacks^19^. Contrarily, reductions in AS within eight weeks to six months have been observed in response to cognitive behavior treatment and pharmacotherapy^20–24^. This literature indicates that in adults AS can change rapidly in highly stressful or treatment conditions, but little is known about the persistence of such changes over time. Moreover, the literature that describes AS as akin to trait anxiety would suggest that AS is relatively stable in the absence of perturbations. Hitherto, the literature lacks an estimate of AS stability over more than twelve months in healthy adults and those with mood symptoms, and this paper was aimed to fill this gap.

In the current study we aim to test the two-year stability of AS and analyze whether a longitudinal association exists between a change in AS and change in severity of anxiety symptoms. As the values of AS and severity of anxiety symptoms measured at consecutive time points are correlated, statistical analysis of longitudinal associations should account for dependencies between these data and adjust for within-subject correlations. This paper is the first to apply such methods to this question. Using generalized estimating equations (GEE) analysis, our models were fit in a heterogeneous sample of participants with a diagnosed anxiety disorder, a depressive disorder, or comorbid anxiety-depressive disorder, as well as participants with no current or lifetime history of an anxiety or depressive disorder. By doing so, the current study extends the previous studies on AS by using a large and heterogeneous sample of participants from various settings and stages of psychopathology. The objectives of the current study are to examine: i) the stability of AS over a two-year time period; ii) whether a change in AS is associated with a concurrent change in severity of anxiety symptoms.

## Materials and Methods

### Study sample

The Netherlands Study of Depression and Anxiety (NESDA) is a national study designed to investigate the course and consequences of depressive and anxiety disorders. At baseline the study included 2981 participants with a mean age of 41.86 years (SD = 13.08, range 18–65 years; 66.39% women), including participants with a past or current depressive and/or anxiety disorder diagnosis (n = 2329; 78.12%) and participants without such a diagnosis (n = 652; 21.87%). To represent various settings and stages of psychopathology, participants were recruited in the general population (n = 564; 18.91%), in general practices (n = 1610; 54.01%), and in mental health organizations (n = 807; 27.07%). Excluded were participants with a primary psychotic, obsessive-compulsive, bipolar or severe addiction disorder and those not being fluent in Dutch. More details of the NESDA study, its design and attrition rates have been described elsewhere^25,26^. For the current study, NESDA data from baseline and the two-year follow-up were used. The investigation was carried out in accordance with the Declaration of Helsinki. The research protocol was approved by the Medical Ethics Review Board (METc) of the VU University Medical Centre Amsterdam^27^, the Medical Ethics Review Board (METc) of the University Medical Center Groningen^28^, and the Medical Ethics Review Board (METc) of the Leiden University Medical Center^29^, and subsequently by local review boards of each participating center. Written informed consent was obtained from all respondents.

### Procedure

At baseline and at two-year follow-up, 1-month diagnoses of anxiety (social anxiety disorder, panic disorder with and without agoraphobia, agoraphobia, and generalized anxiety disorder) and depressive disorders (dysthymia and major depressive disorder) were established with the highly reliable and valid Composite International Diagnostic Interview (CIDI, version 2.1) based on the DSM-IV^30^. The interviews were conducted by a specially trained clinical research staff. The research assistants, who performed the CIDI interviews at follow-up, were blind for baseline diagnoses.

As anxiety symptoms are not limited to anxiety disorders, but are also common in other affective disorders, including depression^7^ and individuals without an affective disorder diagnosis^31^ we included participants with an anxiety disorder diagnosis (at least one current anxiety disorder), a unipolar depressive disorder diagnosis, or a comorbid anxiety-depressive disorder diagnosis, as well as participants without a current or lifetime anxiety or depressive disorder. Excluded were participants who had neither AS nor severity of anxiety symptoms data at both baseline and two-year follow-up (*n* = 10), resulting in a sample of *N* = 2052 available for statistical analysis.

### Self-report questionnaires

#### Anxiety sensitivity

At baseline and at two-year follow-up AS levels were assessed with the Anxiety Sensitivity Index (ASI). The ASI is a self-report questionnaire, consisting of 16 items. The ASI quantifies the fear of anxiety-related symptoms and the concerns about negative consequences of anxiety symptoms^32^. Participants were asked to what extent the items applied to them at the time of measuring. The ASI uses a 5-point Likert scale, ranging from 1 (very little) to 5 (very much). The ASI is scored by summing the ratings for all of the 16 items to obtain a total score that can range from 16 to 80, and can be used as one dimension^33^. The ASI has high internal consistency, adequate test-retest reliability, and good validity^32,34^. Participants with a missing observation at one of the assessment waves were included in the analyses (baseline n = 469, 22.86%; two-year follow-up n = 498, 24.27%), as the planned statistical analysis can adequately handle missing data.

#### Severity of anxiety symptoms

Severity of anxiety symptoms (further referred to as anxiety severity) was determined using the Beck Anxiety Inventory (BAI). The BAI is a self-report instrument with 21 items that assesses the overall severity of anxiety symptoms over the past week on a 4-point Likert scale, ranging from 0 (not at all) to 3 (severely, I could barely stand it). The BAI has shown to have high internal consistency, and good test-retest reliability^35^. The total BAI sum score ranges from 0 to 63. Participants with a missing observation at one of the assessment waves were included in the analyses (baseline n = 11, 0.54%; two-year follow-up n = 348, 16.96%), as the planned statistical analysis can adequately handle missing data.

#### Covariates

Years of education was included as time-independent covariates^36^. This also applied to age and gender^37,38^. Also the reported duration of anxiety symptoms at baseline was included, since this associates with a more severe and chronic course of anxiety and depressive disorders^38,39^. Duration of anxiety symptoms corresponds to the number of months with anxiety symptoms of at least mild severity in the four years prior to baseline, assessed with Life Chart Interview (LCI). The LCI is a standardized interview to retrospectively determine the presence and severity of anxiety and avoidance symptoms^40^. Finally, to account for possible treatment effects, the time-dependent covariates receiving psychological treatment in the last six months before assessment (yes or no, based on Trimbos/iMTA questionnaire for Costs associated with Psychiatric Illness (TIC-P)^41^, and use of antidepressants (50% of the days of a month or more often; yes or no) were included.

### Data analysis

Descriptive statistics were used to present the baseline characteristics of the study sample. Asmundson *et al*.^33^ evaluated the structure of the ASI and found evidence that the latent structure of anxiety sensitivity is dimensional. Therefore, the ASI was used as a continuous measure. Distributions of AS and anxiety severity were checked and found to deviate from a normal distribution.

The changes of AS and anxiety severity scores from baseline to two-year follow-up were tested with a paired samples *t*-test. In samples of our size this *t*-test is valid independent of the distribution of the data^42^. In order to improve on comparability, effect sizes were given as Cohen’s *d*. Effect sizes of 0.20 were considered as small, 0.50 as moderate, and 0.80 as large^43^. Cross-sectional correlations between AS and anxiety severity at baseline and at 2-year follow-up were calculated with Spearman’s rho. The stability of AS was tested with a Spearman’s correlation test. We classified correlations (*r*) as very weak if between 0.00–0.29, weak between 0.30–0.49, moderate between 0.50–0.69, strong between 0.70–0.89, and very strong from 0.90 onwards^44^.

Prior to conducting the longitudinal analyses, multicollinearity for all variables was checked by calculating Spearman correlations and the variance inflation factors (VIF). A Spearman correlation above 0.80 and VIF values above 10 were considered as indicative of severe collinearity^45,46^. The highest Spearman correlation was 0.74. The VIF values of all variables were between 1.00 and 1.62. This indicates that all variables could be maintained in the study without multicollinearity affecting the outcomes too much.

When testing the longitudinal associated changes in AS and changes in anxiety severity over the two-years follow-up time we had to take into account the fact that the data of the two measurements are correlated. The generalized estimating equations (GEE) analysis is a regression analysis technique that takes account of the dependencies between data and adjusts for the within-subject correlations, which makes this technique very suitable for the analysis of longitudinal data. Furthermore, GEE can handle data with a non-normal distribution^47^ In the GEE analyses, AS (independent variable) and anxiety severity (dependent variable) were analyzed simultaneously at both measurement waves to determine the development of AS and anxiety severity over time. A regression coefficient was estimated which reflects the longitudinal association between the change in AS and the change in anxiety severity. Missing data were not imputed as GEE can take these adequately into account^48,49^.

First, a univariable GEE analysis was conducted with AS and anxiety severity. Second, a multivariable GEE analysis was conducted in which the covariates were entered to the model. Gender, age, years of education, and duration of anxiety symptoms at baseline were treated as time-independent factors, whereas psychological treatment and frequent use of antidepressants were analyzed as time-dependent factors.

A p-value < 0.05 was considered to indicate statistical significance. The statistical analyses were performed in IBM SPSS version 23.0 (IBM SPSS statistics for windows, version 23.0., 2013)^50^.

## Results

### Descriptives

Characteristics of the study sample at baseline are summarized in Table 1. Of the whole sample, 652 participants (31.77%) reported no current or lifetime disorder, and 1400 participants (68.23%) had an anxiety and/or depressive disorder diagnosis. Almost half of the participants (46.20%) received psychological treatment and 22.12% used antidepressants. It should be noted that 494 participants (75.77%) in the group without current or lifetime DSM diagnosis, mentioned anxiety symptoms. In addition, 51 participants without current of lifetime diagnosis (7.82%) reported some kind of psychological treatment and 5 participants (0.77%) used antidepressants. Additional information about the baseline characteristics of the separate diagnosis groups and the group without a current or lifetime diagnosis is given in the Supplementary materials Table S1.

**Table 1:** Baseline characteristics for the study sample (*N* = 2052). Means (SD) are given unless stated otherwise.

Baseline characteristics	Mean (SD)/*n (%)*
*Sociodemographics*	
Age, in years	41.64 (13.10)
Female gender, *n (%)*	1330 (64.81)
Education in years	12.00 (3.27)
*Diagnosis*	
Anxiety disorder, *n (%)*	558 (27.19)
Depressive disorder, *n (%)*	307 (14.96)
Comorbid anxiety-depressive disorder, *n (%)*	535 (26.07)
No current or lifetime diagnosis, *n (%)*	652 (31.77)
*Anxiety symptoms*	
Duration with anxiety symptoms (LCI), in number of months	24.73 (19.54)
Anxiety Sensitivity (ASI)	30.20 (10.05)
Severity of anxiety symptoms (BAI)	13.88 (11.52)
*Treatment*	
Receiving psychological treatment, last 6 months, n (%)*	948 (46.20)
Current frequent** use of antidepressants, n (%)*	454 (22.12)

Note: BAI = Beck Anxiety Inventory; LCI = Life Chart Interview; *Combinations of treatments occur; **Frequent ≥ 50% of the days of a month (further details are provided in the method section).

AS decreased from baseline to two-year follow-up (30.02 ± 9.95 vs. 27.66 ± 8.51; t(1448) = 12.75, *p* < 0.001) with a moderate effect size (Cohen’s *d* = 0.34). Next, to facilitate the interpretation of the planned longitudinal association between change in AS and change in anxiety severity, the change of anxiety severity over two-year follow-up was also tested. Anxiety severity decreased (12.95 ± 10.98 vs. 9.60 ± 9.21; t(1693) = 17.34, *p* < 0.001). The effect size was moderate (Cohen’s *d* = 0.43).

### Cross-sectional and temporal correlations

Correlations between all study variables are provided in Supplementary Table S2. AS and anxiety severity showed moderately positive cross-sectional associations at baseline (*r* = 0.67, *p* < 0.001), and at follow-up (*r* = 0.58, *p* < 0.001). Change in AS over the two year interval showed very weak associations with all other variables (*r* = 0.06 to 0.25; *p* < 0.05 to *p* < 0.001). The test-retest correlations of AS (*r* = 0.72, *p* < 0.001) and anxiety severity (*r* = 0.74, *p* < 0.001) were large, indicating substantial stability.

### Longitudinal associations

All longitudinal associations between the changes in AS and changes in anxiety severity are provided in Table 2. The univariable analysis showed that a decrease in AS was associated with a decrease in anxiety severity (*p* < 0.001). This result means that a change of one point of AS over the two-year time period is associated with a change of 0.64 in anxiety severity. In the multivariable analysis, which included all covariates, the longitudinal association between the change in AS and change in anxiety severity remained statistically significant (*p* < 0.001), although the estimate for B decreased slightly.

**Table 2:** Longitudinal associations between change in anxiety sensitivity (AS) and change in severity of anxiety symptoms (anxiety severity), analyzed with generalized estimating equations.

	Severity of anxiety symptoms		
	B	95% CI	*p*
***Univariable***			
AS	**0**.**64**	**0**.**60; 0**.**68**	**<0**.**001**
***Multivariable***			
AS	**0**.**52**	**0**.**48; 0**.**57**	**<0**.**001**
Age	0.02	−0.02; 0.05	0.29
Female gender	0.43	−0.49; 1.35	0.36
Education in years	**−0**.**33**	**−0**.**46; −0**.**19**	**<0**.**001**
Duration with anxiety symptoms (LCI), in number of months	**0**.**06**	**0**.**04; 0**.**09**	**<0**.**001**
Receiving psychological treatment, last 6 months	**−2**.**34**	**−3**.**10; −1**.**57**	**<0**.**001**
Current frequent **use of antidepressants, n (%)*	**−1**.**69**	**−2**.**57; −0**.**81**	**<0**.**001**

Note: Univariable analyses: AS = independent factor; Multivariable analyses: AS = independent factor adjusted for the time-independent covariates gender, age, years of education, and duration of anxiety symptoms at baseline and the time-dependent covariates psychological treatment and frequent use (i.e. ≥50% of the days of a month) of antidepressants. *Combinations of treatments occur; **Frequent ≥ 50% of the days of a month (further details are provided in the method section). Values in bold indicate statistical significance.

Post-hoc analyses were performed on the physical and social-cognitive subscales of the ASI-instrument. Results are described in the text, and tables are shown in the Supplementary materials S3 and S4.

Some studies suggested that specific AS subfactors accounted for associations between AS and anxiety or mood^51,52^, whereas all AS factors have been implicated in suicidal ideation^14^. Post-hoc we tested whether the association between AS and anxiety severity was primarily driven by physical or by social-cognitive factors, using the physical and social-cognitive AS subscales of the ASI instrument^51^. Repeating the analyses with these two subscales separately showed that the results were comparable to those described above for the full ASI scale (see Supplementary materials S3 and S4), which suggests that both factors play a role in the link with severity of anxiety symptoms.

## Discussion

This study in this large and heterogeneous cohort of adult participants with and without a diagnosed anxiety and/or depressive disorder, yielded two key observations. First, AS showed a strong two-year stability with a moderate decrease over time that was in the same range as the decrease in severity of anxiety symptoms over time. Second, the decrease in AS was positively associated with a decrease in severity of anxiety symptoms.

We found a combination of high rank-order consistency and moderate decrease in mean-level AS scores. These statistics can be interpreted incorrectly. It may seem that AS scores of all participants would have decreased to the same extent, yet the significant mean-level change explained only a marginal part of the two-year test-retest stability of AS (i.e. *d* = 0.34 translates roughly to *r* = 0.17, thus about 3% explained variance (R^2^) in AS at follow-up), which cannot be considered of much importance to our interpretations of AS stability. Although AS proved to be a highly stable characteristic, the test-retest estimate also leaves room for potentially substantial changes in some individuals as well^53^. A combination of genetic factors, which are generally stable over time, and environmental factors, which can be time-specific, could be the cause of this result, as indicated by Zavos *et al*.^18^ in a study AS in adolescent twins.

The comparable decreases in AS and severity of anxiety symptoms over the two-year interval, in combination with the positive association between these changes suggests that the two constructs are connected but not necessarily similar. There are important conceptual differences; whereas severity of anxiety symptoms refers to the extent to which actual symptoms are perceived, AS refers to a misinterpretation of these symptoms, namely the belief that anxiety symptoms are harmful and will have catastrophic consequences. This misinterpretation can potentially lead to more and more severe anxiety symptoms^54^. In the multivariate analyses, where potential confounding influences were taken into account, both psychological treatment and the use of antidepressants were associated with a change in severity of anxiety symptoms (Table 2). However, correlations indicated that psychological and pharmacological treatment were not associated with a decrease in AS (Supplementary materials). These findings underline that AS and severity of anxiety symptoms are two different constructs. In line with previous studies^4,20,55^, the results of our study support the conclusion that AS can be conceived as a more generic psychological construct directly related to severity of anxiety symptoms. It has been suggested that the link between AS and anxiety and mood are driven by the cognitive aspects of AS^52,56^, but our post-hoc analyses indicated that both physical and social-cognitive AS factors were implicated in changes in the severity of anxiety symptoms, in line with a recent meta-analyses that linked all AS factors to suicidal ideation^14^.

The longitudinal association between a change in AS and a change in severity of anxiety symptoms is interesting from a clinical perspective, as a core treatment intervention for individuals suffering from panic attacks is interoceptive exposure^57^. This approach targets AS by deliberately bringing on physical sensations that are harmless, yet feared. For example, an individual with a panic disorder might be instructed to undergo a hyperventilation provocation exercise in order to make his or her heart rate speed-up or feel dizzy, and therefore learn that these sensations are not dangerous. It would be worthwhile to examine whether intervening on AS is beneficial not only for individuals suffering from panic attacks, but for all individuals who suffer from anxiety symptoms. An indication that a treatment focused on AS can be successful is reported by Watt and Stewart^58^, and by Schmidt *et al*.^22^. The first study showed that a treatment program aiming at reducing AS can also reduce anxiety and depressive symptoms, and the latter showed that a computerized treatment can reduce elevated AS levels in patients with severe pathology and suicidal ideation. The findings of these studies are also important because a recent meta-analysis showed that AS was associated with suicidal thoughts and behaviors^14^. In sum, treatment aiming at reducing an individual’s AS level may have an additional advantage as it diminishes the risk of future development of anxiety psychopathology and anxiety disorders and even of other Axis I disorders^59^.

The results of this study expand the literature on the stability of AS over time in adults. The second aim was to analyze the associations between changes in AS and changes in severity of anxiety symptoms. Strengths of this study include its large heterogeneous sample size, the broad range of repeated assessments of psychopathology, and the fact that participants were recruited from various settings and with different stages of disorder. At the same time, the results are subject to some limitations. First, since AS levels were previously found to differ between different anxiety disorder diagnoses^55^, the association between changes in AS and severity of anxiety symptoms may differ between the different anxiety disorder groups. In analogy, this might also apply to the different depressive disorders. However, given the high comorbidity between different anxiety disorders^60^, the low temporal stability of anxiety disorder diagnoses^61,62^, and the high comorbidity rates between anxiety and depressive disorders^63,64^, we believe that in our longitudinal model such analyses would not provide additional information to the current study. Second, assessment of AS and severity of anxiety symptoms relies on self-report measures. Although these instruments were proven to be reliable and valid, they might be subject to social desirability and recall bias^65^. Third, it should be noted that our finding that AS is associated with severity of anxiety symptoms does not infer causality, which requires studies of individual level processes and interventions^66^. Fourth, AS was tested with the original ASI^32^. The more recently developed ASI-3 appears to have better psychometric properties^67^, but within NESDA, the original ASI has been used to enable longitudinal analysis. Fifth, it would be interesting to study processes related to AS with more frequent assessments, as this higher time resolution may help further unravel the dynamics of stability and change in AS. Future studies of individual differences in AS stability and change processes are needed^66^, next to normative change^68^. However, these kinds of analyses are beyond the scope of this study. Finally, our analyses do not contribute much to the debate about the overlap between AS and trait-anxiety (see introduction), although the stabilities we observed for AS seem somewhat lower^69^. Future studies may examine how the stability of AS compares to the stability of trait anxiety and other related psychological constructs such as neuroticism, rumination, and worry, as well as their overlap and incremental value.

## Conclusion

The stability of AS is strong and comparable to related psychological trait constructs. Decreases in AS are associated with decreases in severity of anxiety symptoms. This result may imply that intervening on AS may be beneficial for all individuals who are suffering from anxiety symptoms. Future research is recommended to establish whether a treatment focused at reducing AS is of added value for reducing current anxiety symptoms as well as long-term relapse prevention effects.

## Supplementary information

Supplementary materials

## Data Availability

The datasets analyzed during the current study are available upon reasonable request from NESDA, Amsterdam: nesda@ggzingeest.nl.
