# Supplementary material for: Anxiety sensitivity, its stability and longitudinal association with severity of anxiety symptoms
Source: Sci Rep. 2019 Mar 13;9:4314. doi: 10.1038/s41598-019-39931-7 (PMC6416311; doi:10.1038/s41598-019-39931-7)
Supplement: Supplementary file 1 — Supplementary materials [file 41598_2019_39931_MOESM1_ESM.pdf]

Supplementary materials

Anxiety sensitivity, its stability and longitudinal association with severity of anxiety symptoms

Johanna HM Hovenkamp-Hermelink[1], Date C van der Veen[1], Richard C Oude Voshaar[1], Neeltje M Batelaan[2,3], Brenda Penninx[2,3], Bertus F. Jeronimus[1,4], Robert Schoevers[1], Harriëtte Riese[1]

[1] University of Groningen, University Medical Center Groningen (UMCG), Department of Psychiatry, Interdisciplinary Center Psychopathology and Emotional regulation (ICPE), Groningen, The Netherlands

[2] VU University Medical Center Amsterdam, Department of Psychiatry and Amsterdam Public Health Research Institute, Amsterdam, The Netherlands

[3] GGZ inGeest Amsterdam, the Netherlands

[4] University of Groningen, Department of Developmental Psychology, Faculty of Behavioral and Social Sciences, University of Groningen, Groningen, The Netherlands

Table S1. Baseline characteristics for the study sample (*N* = 2052). Means (SD) are given unless stated otherwise

	Total sample N=2052	Anxiety disorders N=558 (21.19%)	Depressive disorders N=307 (14.96%)	Comorbid anxiety-depressive disorders N=535 (26.07%)	Healthy controls N=652 (31.77)
Sociodemographics					
Age in years	41.64 (13.10)	41.53 (12.49)	43.03 (13.12)	43.17 (11.75)	41.10 (14.44)
Female gender, *n (%)*	1330 (64.81)	377 (67.56)	195 (63.52)	358 (66.92)	400 (61.35)
Education in years	12.00 (3.27)	12.22 (3.29)	11.97 (3.21)	11.41 (3.17)	12.95 (3.29)
Anxiety symptoms					
LCI	24.73 (19.54)	25.94 (20.05)	17.53 (15.76)	31.69 (18.57)	9.65 (13.25)
Anxiety Sensitivity (ASI)	30.20 (10.05)	34.85 (9.82)	31.33 (9.06)	36.63 (10.13)	25.53 (5.84)
Severity of anxiety symptoms (BAI)	13.88 (11.52)	16.39 (9.35)	15.92 (8.56)	22.59 (10.49)	6.57 (5.49)
Treatment					
Receiving psychological treatment, last 6 months, *n (%)**	948 (46.20)	304 (54.48)	207 (67.43)	386 (72.15)	51 (7.82)
Current frequent** use of antidepressants, *n (%)**	454 (22.12)	153 (27.42)	95 (30.94)	201 (35.57)	5 (0.77)

Note: BAI = Beck Anxiety Inventory; LCI = Life Chart Interview; * combinations of treatments occur; ** frequent ≥ 50% of the days of a month (further details in method section of the manuscript)

Table S2. Spearman correlations between the study variables

	Variable	1	2	3	4	5	6	7	8	9	10	11	12	13	14	15	16	17
1	Age	-																
2	Gender	-0.09[c]	-															
3	Years of education	-0.09[c]	-0.02	-														
4	Duration anxiety symptoms. T_0	0.08[b]	-0.04	-0.11[c]	-													
5	AS T_0	0.04	0.04	-0.15[c]	0.25[c]	-												
6	AS T_1	0.09[b]	0.03	-0.06[a]	0.17[c]	0.72[c]	-											
7	Δ AS	-0.08[b]	0.04	-0.12[c]	0.11[b]	0.49[c]	-0.19[c]	-										
8	Anxiety severity T_0	-0.01	0.09[c]	-0.23[c]	0.29[c]	0.67[c]	0.55[c]	0.25[c]	-									
9	Anxiety severity T_1	0.08[b]	0.09[c]	-0.18[c]	0.27[c]	0.55[c]	0.58[c]	0.08[b]	0.74[c]	-								
10	Psychol. treatment T_0	-0.15[c]	0.01	-0.08[c]	0.20[c]	0.34[c]	0.26[c]	0.16[c]	0.47[c]	0.32[c]	-							
11	Psychol. treatment T_1	-0.13[c]	0.03	-0.02	0.15[c]	0.30[c]	0.25[c]	0.10[c]	0.41[c]	0.34[c]	0.60[c]	-						
12	Antidepressant use T_0	0.06[b]	-0.00	-0.10[c]	0.14[c]	0.29[c]	0.23[c]	0.12[c]	0.39[c]	0.30[c]	0.43[c]	0.32[c]	-					
13	Antidepressant use T_1	0.06[a]	0.01	-0.08[b]	0.10[c]	0.23[c]	0.21[c]	0.06[a]	0.33[c]	0.28[c]	0.33[c]	0.30[c]	0.64[c]	-				
14	AS-physical T_0	0.06[a]	0.06[a]	-0.14[c]	0.18[c]	0.92[c]	0.66[c]	0.45[c]	0.59[c]	0.49[c]	0.27[c]	0.22[c]	0.23[c]	0.20[c]	-			
15	AS-social-cognitive T_0	0.01	0.00	-0.12[c]	0.26[c]	0.83[c]	0.60[c]	0.40[c]	0.59[c]	0.49[c]	0.34[c]	0.32[c]	0.31[c]	0.24[c]	0.58[c]	-		
16	AS-physical T_1	0.12[c]	0.03	-0.08[b]	0.11[c]	0.65[c]	0.91[c]	-0.17[c]	0.48[c]	0.51[c]	0.20[c]	0.18[c]	0.18[c]	0.18[c]	0.70[c]	0.43[c]	-	
17	AS-social-cognitive T_1	0.02	0.01	-0.03	0.18[c]	0.59[c]	0.82[c]	-0.14[c]	0.48[c]	0.49[c]	0.25[c]	0.26[c]	0.23[c]	0.20[c]	0.43[c]	0.68[c]	0.53[c]	-

T_0 = measurement at baseline; T_1 = measurement at two-year follow-up; AS = Anxiety sensitivity, assessed with the Anxiety Sensitivity Index; Δ AS = change in AS score T_0 - T_1; Anxiety severity = severity of anxiety symptoms, assessed with the Beck Anxiety Inventory; AS-physical = physical subscale of the Anxiety Sensitivity Index; ASI-social-cognitive = social-cognitive subscale of the Anxiety Sensitivity Index;; [a]: $p<0.05$; [b]: $p<0.01$; [c]: $p<0.001$.

1. Anxiety Sensitivity Index subscales

1.1 Methods

Earlier factor analysis performed on The Netherlands Study of Depression and Anxiety (NESDA) baseline ASI data (Drost et al., 2012) revealed a two subscales solution: a *physical* and a combined *social-cognitive* subscale. Two items (7 and 13) were not included in the subscales due to the low loadings on each of the subscales. The ASI uses a 5-point scale, ranging from 1 (very little) to 5 (very much). The *ASI-physical* subscale refers to the fear of somatic sensations and the consequences of it, and includes questions like “It scares me when my heart beats rapidly” or “It scares me when I am short of breath”. It is calculated from 8 items and the summed score can range from 8 to 40. The *ASI-social-cognitive* subscale refers to fear of anxiety symptoms that are publicly observable or the fear of losing mental control; it includes questions like “It is important to me not to appear nervous” or “When I am nervous, I worry that I am mentally ill”. It is calculated from 6 items and the summed score can range from 6 to 30.

1.2. Results

1.2.1. Descriptives of subscale scores

Table S2 presents the scores of the two subscales at baseline and two-year follow-up. The *ASI subscale* scores decreased from baseline to 2-year follow-up ($p < 0.001$). The effect sizes (Cohen’s d) for the changes in the scores were moderate: *ASI-physical* subscale $d = 0.30$ and *ASI-social-cognitive* subscale $d = 0.28$ .These changes over two years and the effect sizes are comparable to the effect size of the ASI-total scores.

1.2.2. Cross-sectional and temporal correlations

The cross-sectional associations between the Beck Anxiety Inventory (BAI) and both ASI-subscale scores were moderately positive (all *p*-values < 0.001) at baseline, as well as at 2-year follow-up. The Spearman correlation between BAI and *ASI-physical* subscale was 0.59 (baseline), and 0.51 (2-year follow-up). For the *ASI-social-cognitive* subscale, the correlations were 0.59 (baseline) and 0.49 (2-year follow-up). The test-retest correlations of AS-physical and ASI-social-cognitive subscales were large (r = 0.70 and r = 0.68, respectively).

1.2.3. Longitudinal associations between ASI-subscales and severity of anxiety symptoms

The longitudinal associations between the ASI subscale scores and the BAI scores are given in Table S3. The results of the subscales were comparable with the results found for the ASI-total scores.

Table S3. Descriptives of the *ASI-physical* and *ASI-social-cognitive* subscales at baseline and two-year follow-up

**Variable**	**N (% of total sample)**	**Baseline mean (SD)**	**2-yr follow-up mean (SD)**	***t*-value**	***p*-value***
**ASI-physical subscale**	1464 (71.34)	14.81 (6.27)	13.48 (5.40)	11.28	<0.001
**ASI-social-cognitive subscale**	1475 (71.88)	12.61 (4.39)	11.70 (3.85)	10.43	<0.001

Note: *paired samples t-test; ASI = Anxiety Sensitivity Index

Table S4. Longitudinal associations between *ASI-physical* subscale (independent), or *ASI-social-cognitive* subscale (independent) scores with anxiety severity (dependent) scores, analyzed with generalized estimating equations

		Severity of anxiety symptoms		
ASI-physical		B	95% CI	*p*-value
	Univariable			
	ASI-physical	**0.89**	**0.83, 0.95**	**<0.001**
	Multivariable			
	ASI-physical	**0.71**	**0.64, 0.78**	**<0.001**
	Age	0.01	-0.02, 0.05	0.440
	Female gender	0.12	-0.85, 1.10	0.805
	Education in years	**-0.34**	**-0.48, -0.20**	**<0.001**
	Duration (number of months) with anxiety symptoms (LCI)	**0.08**	**0.05, 0.10**	**<0.001**
	Receiving psychological treatment, last 6 months	**-2.55**	**-3.33, -1.76**	**<0.001**
	Current frequent use of antidepressants	**-2.12**	**-3.02, -1.21**	**<0.001**
ASI-social-cognitive				
	Univariable			
	ASI-social-cognitive	**1.21**	**1.13, 1.30**	**<0.001**
	Multivariable			
	ASI-social-cognitive	**0.95**	**0.85, 1.04**	**<0.001**

Age	0.03	-0.003, 0.07	0.075
Female gender	0.78	-0.19, 1.75	0.115
Education in years	**-0.41**	**-0.55, -0.27**	**<0.001**
Duration (number of months) with anxiety symptoms (LCI)	**0.07**	**0.05, 0.09**	**<0.001**
Receiving psychological treatment, last 6 months	**-2.31**	**-3.11, -1.50**	**<0.001**
Current frequent use of antidepressants	**-1.61**	**-2.53, -0.69**	**0.001**

Note: ASI = Anxiety Sensitivity Index; *AS-physical* = physical subscale of the Anxiety Sensitivity Index; *ASI-social-cognitive* = social-cognitive subscale of the Anxiety Sensitivity Index;
Univariable analyses: ASI as independent factor;
Multivariable analyses: ASI as independent factor with controlling for the time-dependent covariates psychological treatment and frequent use of antidepressants, and the time-independent covariates gender, age, years of education, and duration of anxiety symptoms at baseline.
Values printed in bold face indicate statistical significance.

References

Drost, J., Van der Does, A. J. W., Antypa, N., Zitman, F. G., Van Dyck, R., & Spinhoven, P. (2012). General, specific and unique cognitive factors involved in anxiety and depressive disorders. *Cognitive Therapy and Research*, *36*(6), 621–633. http://doi.org/10.1007/s10608-011-9401-z
